# Supplementary material for: Number of children and body composition in later life among men and women: Results from a British birth cohort study
Source: PLoS One. 2019 May 29;14(5):e0209529. doi: 10.1371/journal.pone.0209529 (PMC6541250; doi:10.1371/journal.pone.0209529)
Supplement: S1 Table — ** Adjusted model includes childhood cognitive function, childhood and adult socioeconomic position, education and cigarette smoking. (DOCX) [file pone.0209529.s001.docx]

|  |  | Unadjusted model | | | |  | Adjusted model** | | | |  |
| --- | --- | --- | --- | --- | --- | --- | --- | --- | --- | --- | --- |
| **Outcome** | **N** | **Regression coefficients (95% CI)**  **compared with baseline group of 0 children** | | | | **P-Value,**  **Trend** | **Regression coefficients (95% CI)**  **compared with baseline group of 0 children** | | | | **P Value,**  **Trend** |
|  |  | **Number of children** | | | |  | **Number of children** | | | |  |
|  |  | **1** | **2** | **3** | **4+** |  | **1** | **2** | **3** | **4+** |  |
| **Body Mass Index (kg/m2)** |  |  |  |  |  |  |  |  |  |  |  |
| **Maximum Sample** |  |  |  |  |  |  |  |  |  |  |  |
| *Men* | 990 | 0.77  (-0.26,1.80) | 0.48  (-0.27,1.22) | 0.36  (-0.52,1.25) | 1.34 (0.17,2.51) | 0.1 | 0.44  (-0.60,1.47) | 0.40  (-0.34,1.15) | 0.18  (-0.70,1.06) | 1.29 (0.13,2.45) | 0.2 |
| *Women* | 1,117 | 0.44  (-0.91,1.80) | 0.35  (-0.74,1.43) | 1.19 (0.00,2.38) | 1.43  (-0.04,2.91) | 0.02 | 0.15  (-1.23,1.51) | 0.10  (-0.99,1.20) | 0.86  ( -0.33,2.06) | 1.08  (-0.41,2.57) | 0.05 |
| **Sample attending CRF** |  |  |  |  |  |  |  |  |  |  |  |
| *Men* | 731 | 1.01  (-0.21,2.24) | 0.48  (-0.41,1.37) | 0.37  (-0.68,1.41) | 1.39 (0.07,2.71) | 0.2 | 0.63  (-0.61 ,1.87) | 0.47  (-0.42,1.37) | 0.16  (-0.88,1.20) | 1.41 (0.06,2.76) | 0.2 |
| *Women* | 830 | 0.60  (-0.83,2.04) | 0.07  (-1.06,1.20) | 0.67  (-0.58,1.92) | 1.22  (-0.34,2.78) | 0.2 | 0.38  (-1.07,1.83) | -0.06  (-1.21,1.08) | 0.50  (-0.77,1.77) | 0.94  (-0.65,2.53) | 0.3 |
| **Waist Circumference (cm)** |  |  |  |  |  |  |  |  |  |  |  |
| **Maximum Sample** |  |  |  |  |  |  |  |  |  |  |  |
| *Men* | 990 | 0.99  (-1.80,3.78) | 0.39  (-1.63,2.42) | 0.49  (-1.91,2.88) | 2.87  (-0.28,6.02) | 0.2 | -0.07  (-2.84,2.70) | 0.16  (-1.86,2.18) | 0.02  (-2.35,2.39) | 2.49  (-0.65,5.63) | 0.3 |
| *Women* | 1,115 | 1.56  (-1.66,4.79) | 0.61  (-1.97,3.19) | 1.30  (-1.53,4.13) | 4.34 (0.82,7.86) | 0.06 | 0.81  (-2.43,4.05) | -0.07  (-2.68,2.53) | 0.44  (-2.41,3.30) | 3.57  (-0.01,7.12) | 0.2 |
| **Sample attending CRF** |  |  |  |  |  |  |  |  |  |  |  |
| *Men* | 730 | 1.39  (-1.88,4.67) | 0.53  (-1.84,2.90) | 0.41  (-2.38,3.19) | 3.40  (-0.13,6.94) | 0.3 | 0.25  (-3.08 ,3.57) | 0.31  (-2.09,2.71) | -0.13  (-2.93,2.68) | 3.01  (-0.53,6.71) | 0.3 |
| *Women* | 828 | 2.04  (-1.46,5.55) | 0.28  (-2.50,3.04) | 1.14  (-1.92,4.20) | 4.26 (0.42,8.08) | 0.1 | 1.53  (-2.03,5.10) | -0.02  (-2.84,2.80) | 0.77  (-2.34,3.88) | 3.81  (-0.13,7.74) | 0.2 |
